# Association between hepcidin levels and inflammatory bowel disease: A systematic review and meta‐analysis of observational studies

**DOI:** 10.1002/fsn3.4146

**Published:** 2024-04-08

**Authors:** Samira Soltanieh, Marieh Salavatizadeh, Mihnea‐Alexandru Gaman, Hamed Kord Varkaneh, Shing Cheng Tan, Kousalya Prabahar, Oana Deliu Lozovanu, Heitor O. Santos, Azita Hekmatdoost

**Affiliations:** ^1^ Department of Clinical Nutrition and Dietetics, Faculty of Nutrition and Food Technology Shahid Beheshti University of Medical Sciences Tehran Iran; ^2^ Faculty of Medicine, "Carol Davila" University of Medicine and Pharmacy, Bucharest, Romania & Department of Hematology, Centre of Hematology and Bone Marrow Transplantation Fundeni Clinical Institute Bucharest Romania; ^3^ Department of Nutrition and Food Hygiene, Nutrition Health Research Center Hamadan University of Medical Sciences Hamadan Iran; ^4^ UKM Medical Molecular Biology Institute Universiti Kebangsaan Malaysia Kuala Lumpur Malaysia; ^5^ Department of Pharmacy Practice, Faculty of Pharmacy University of Tabuk Tabuk Saudi Arabia; ^6^ University of Normandie Caen France; ^7^ School of Medicine Federal University of Uberlandia (UFU) Uberlandia Brazil

**Keywords:** anemia, hepcidin, inflammatory bowel disease, iron homeostasis, prohepcidin

## Abstract

Hepcidin has a crucial role in iron homeostasis upon inflammatory conditions such as inflammatory bowel disease (IBD). Thus, we conducted a systematic review and meta‐analysis to determine the overall association between serum hepcidin concentrations and IBD. Based on the preferred reporting items for systematic review and meta‐analysis (PRISMA) protocols, an electronic literature search was conducted on PubMed/MEDLINE, Scopus, and Web of Science until June 2020. Studies were deemed eligible for inclusion if they met the following criteria: (1) diagnosis of IBD, (2) observational design, and (3) measured serum hepcidin and prohepcidin concentrations in IBD patients and control group. Overall, 10 studies including 1184 participants were evaluated. Random‐effects meta‐analysis revealed that subjects with IBD had 7.22 ng/mL (95% CI: 2.10, 12.34; *p* = .006) higher serum hepcidin concentrations compared to control groups. A nonsignificantly lower serum prohepcidin concentration (0.522 ng/mL, 95% CI: −1.983 to 0.939; *p* = .484) was found for IBD patients compared to healthy subjects. However, there was significant heterogeneity among the studies regarding both hepcidin (*I* ^2^ = 98%, *p* < .001) and prohepcidin levels (*I* ^2^ = 96%, *p* < .001), respectively. In an age‐based subgroup analysis, patients aged ≥18 years with IBD displayed higher serum hepcidin levels when compared to healthy individuals (22.36 ng/mL, 95% CI, 2.12–42.61; *p =* .030). Hepcidin concentrations are elevated in subjects with IBD; however, the clinical relevance of this finding requires further evaluation in future investigations as the increase is relatively small compared to the wide range of normal hepcidin values.

## Graphical abstract

Subjects with IBD had 7.22 ng/mL higher serum hepcidin concentrations compared to control groups. Lower serum prohepcidin concentration was found for IBD patients compared to healthy ones.

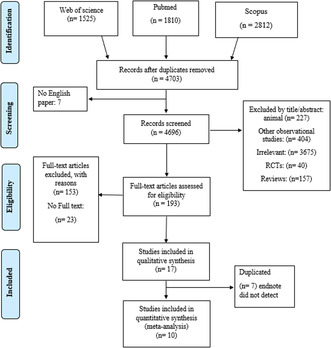

## 1 INTRODUCTION

Inflammatory bowel disease (IBD) is a chronic idiopathic inflammatory disorder of the gastrointestinal tract with relapsing periods, comprising the phenotypes of ulcerative colitis (UC) and Crohn's disease (CD) (Dong et al., 2017). Several studies have reported a multifactorial etiology for IBD, and it is argued that the combination of genetic, environmental, and immunological factors can lead to a defect in gut microflora and activation of immune system responses (Abraham & Cho, 2009; Cheifetz, 2013; Kühl et al., 2015; Moura et al., 2015).

Nevertheless, several investigations have hypothesized that there may be a role in IBD pathophysiology for hepcidin, an antimicrobial 25‐amino acid peptide produced mostly in hepatocytes with a fundamental role in iron homeostasis (Arnold et al., 2009; Krawiec et al., 2017; Oustamanolakis et al., 2011). Hepcidin binds to ferroportin and inhibits iron transfer from the basolateral membrane of erythrocytes to blood circulation, thus resulting in impaired erythropoiesis (Katsarou & Pantopoulos, 2018). Hepcidin expression can be regulated by iron status and inflammation (Wang & Babitt, 2016). Proinflammatory cytokines, especially interleukin‐6 (IL‐6), high iron stores, and infections induce hepcidin synthesis whereas low iron stores, anemia, and hypoxia are linked to suppressor effects (Wang & Babitt, 2016). In this sense, hepcidin and related inflammatory cytokines play a pivotal role in the pathogenesis of anemia in IBD (Aksan et al., 2019). However, whether nature of the association between hepcidin and IBD remains controversial, with some investigations suggesting higher serum hepcidin concentrations in both UC and CD versus healthy individuals (Oustamanolakis et al., 2011), whereas others have depicted low hepcidin levels in children and adult with IBD (Arnold et al., 2009; Krawiec et al., 2017). Therefore, the aim of this study was to ascertain the overall extent to which serum hepcidin levels differ between IBD patients and healthy controls.

## 2 METHODS

This systematic review and meta‐analysis was performed based on the preferred reporting items for systematic review and meta‐analysis (PRISMA) protocol.

### 2.1 Search strategy

Electronic literature search was conducted in PubMed/MEDLINE, Scopus, and Web of Science until June 2020. In order to find pertinent articles, we searched using the following keywords using the medical subject heading (MeSH) and non‐MeSH terms: (“inflammatory bowel diseases”[Mesh] OR “inflammatory bowel disease”[tiab] OR “colitis, ulcerative”[Mesh] OR “ulcerative colitis”[tiab] OR **“**Crohn disease”[Mesh] OR “Crohn**'**s disease”[tiab] OR “IBD”[tiab] OR “colitis”[Mesh] OR “colitis”[tiab] OR “inflammatory bowel disorder”[tiab] OR “Gastroenteritis”[Mesh] OR “Gastroenteritis”[tiab]) AND (“Hepcidins”[Mesh] OR “Hepcidin”[tiab] OR “iron”[Mesh] OR “iron”[tiab] OR “serum iron”[tiab] OR “ferritins”[Mesh] OR “ferritin”[tiab] OR “iron homeostasis”[tiab] OR “iron deficiency”[tiab] OR “Prohepcidin”[tiab]). There was no restriction on publication time and language. For more confidence, we scanned the reference lists of included studies and reviews. Unpublished records and gray literature were not included in this review.

### 2.2 Eligibility criteria

Studies were included in this review if they met the following criteria: (1) clinically diagnosed IBD patients of any age and sex; (2) observational studies; (3) studies that measured serum hepcidin and prohepcidin concentrations in IBD patients versus controls. Animal studies, case reports, literature reviews, and studies published in a language other than English were excluded.

### 2.3 Data extraction and quality assessment

Relevant data were independently extracted by two authors (S.S and M.S.). Any disagreements between the two reviewers were resolved by a third reviewer (H.K.V.). Extracted data included the main outcomes (means and SDs of serum hepcidin and prohepcidin levels), first author name, publication year, study location, study design, participants' mean age and gender, and case and control groups. We assessed the quality of relevant studies based on the Newcastle–Ottawa Scale (NOS) which comprises the following criteria: (1) adequate definition of case, (2) representativeness of cases, (3) selection of control, (4) definition of control, (5) control for important factor or additional factor, (6) exposure assessment, (7) same method of ascertainment for cases and controls, (8) nonresponse rate. Finally, an overall score of 0–8 was given to studies and they were classified as low, moderate, and high quality (Wells et al., 2000).

### 2.4 Statistical analysis

All analyses were carried out in the Stata software (version 14) and *p‐*values less than 0.05 were considered statistically significant. Der Simonian and Laird's random‐effects model was used to conduct the meta‐analyses and calculate the pooled effect size of the mean serum hepcidin levels in IBD patients (Dersimonian & Laird, 1986). The overall relationship between the serum hepcidin levels and IBD was estimated by using the mean and standard deviation (SD) for serum hepcidin concentrations in both IBD and control groups. Sensitivity analyses were carried out by the metaninf test to assess the impact of each study on the overall result. Funnel plots and Egger's linear regression tests were performed to investigate the publication bias (Egger et al., 1997). In order to determine possible sources of heterogeneity, we conducted subgroup analyses. Cochran's *Q* test and the *I* ^2^ statistic were used to test the heterogeneity between the included studies (Higgins & Thompson, 2002).

## 3 RESULTS

### 3.1 Search results and study characteristics

**FIGURE 1:**
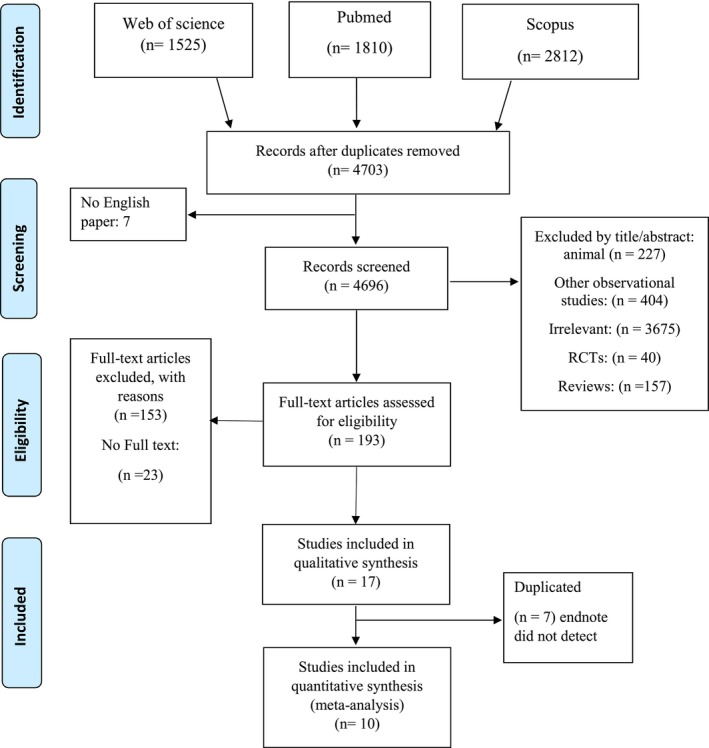
Flow diagram of the number of studies entered into the meta‐analysis.

Overall, 6147 articles were identified in PubMed/MEDLINE, Scopus, and Web of Science databases of which 1444 duplicate articles were excluded. After screening the titles and abstracts of the 4703 remaining articles, 193 publications' full texts were assessed for eligibility of which 153 studies were excluded due to lack of data of interest, 23 studies were excluded due to no full‐text availability, and seven papers were excluded for being duplicates. Finally, this systematic review and meta‐analysis was conducted on 10 articles (Figure 1). Characteristics of eligible studies are summarized in Table 1. Included studies were published between 2009 and 2019 and conducted in Israel (Moran‐Lev et al., 2019), Hungary (Nagy et al., 2010), Greece (Oustamanolakis et al., 2011), UK (Arnold et al., 2009), Italy (Bergamaschi et al., 2013; Martinelli et al., 2016), Turkey (Kaya et al., 2011), Poland (Krawiec et al., 2017), Switzerland (Mecklenburg et al., 2014), and Czech Republic (Karaskova et al., 2018). Sample sizes varied from 33 (Kaya et al., 2011) to 268 (Mecklenburg et al., 2014) participants. All of the studies encompassed both sexes.

**TABLE 1:** Characteristics of included studies.

Study, year	Country	Study design	Sex	*N*	Mean age		Type of IBD	Disease activity	IDA (*n*)	ACD (*n*)	IDA + ACD (*n*)	Outcomes	Samples/method of quantification	Newcastle–Ottawa scale
					Case	Control								
Moran‐Lev et al. (2019)	Israel	Prospective	Both	85	13.5	12.3	CD and UC	Active, moderate to severe or mild–moderate forms; PCDAI: 39.8 ± 14.4 (15–70); PUCAI 30.8 ± 8.3 (20–50)	NR	NR	NR	Hepcidin	Serum /ELISA	5
Nagy et al. (2010)	Hungary	Case–control	Both	130	NR		CD and UC	CDAI: 96.30 ± 44.49; UCAI: 141.09 ± 86.91	NR	NR	NR	Prohepcidin	Serum/ELISA	7
Oustamanolakis et al. (2011)	Greece	Case–control	Both	202	24.52	21.15	CD and UC	CDAI >150 points = active (*n* = 9), <150 points = inactive (*n* = 42); SCCAI >3 points = active (*n* = 11), <3 points = inactive (*n* = 38)	NR	NR	NR	Hepcidin/Prohepcidin	Serum/ELISA	8
Arnold et al. (2009)	United Kingdom	Case–control	—	86	44.03	36.02	CD and UC	Active and quiescent disease; Median Harvey–Bradshaw index (CD) = 6; median simple clinical colitis activity index (UC) = 8	18	2	NR	Hepcidin	Blood samples/radioimmunoassay	6
Bergamaschi et al. (2013)	Italy	Case–control	Both	90	46	43	CD and UC	Active disease (*n* = 26), quiescent disease (*n* = 28); Active disease = CDAI >150 for CD, CCAI >4 for UC	9	7	4	Hepcidin	Serum/surface‐enhanced laser desorption/ionization time‐of‐flight mass spectrometry	6
Kaya et al. (2011)	Turkey	Case–control	Both	33	9.6	8.8	NR	Disease activity evaluated by ESR, CRP	NR	NR	NR	Prohepcidin	Serum/ELISA	4
Krawiec et al. (2017)	Poland	Case–control	Both	96	12.96	11.2	CD and UC	UC: active (*n* = 28), in remission (*n* = 18), median MTWAI = 4.5 points; CD: active (*n* = 26), in remission (*n* = 3), median PCDAI 30 points	NR	NR	NR	Hepcidin	Serum/ELISA	7
Martinelli et al. (2016)	Italy	Cross‐sectional	Both	98	12.6	11.1	CD and UC	PUCAI 13.8 ± 16.4 (0–47.5); PCDAI 11.8 ± 14.4 (0–60)	8	2	12	Hepcidin	Serum/mass spectrometry‐based assay	7
Mecklenburg et al. (2014)	Switzerland	Case–control	Both	247	18.85	—	CD and UC	CD: active disease (CDAI >150, CRP > 5), *n* = 35; inactive disease (CDAI ≤150, CRP ≤5), *n* = 70; UC: active disease (MTWAI ≥5, CRP >5), n = 60; Inactive disease (MTWAI ≤5, CRP ≤5), n = 57	NR	NR	NR	Hepcidin	Serum/ELISA	6
Karaskova et al. (2018)	Czech Republic	Cross‐sectional	Both	96	13.17	12.1	CD and UC	PCDAI: 36.3 (28.8–45.0) points; PUCAI: 30.0 (20.0–45.0) points	11	28	NR	Hepcidin	Serum/reverse‐phase liquid chromatography	6

Abbreviations: ACD, anemia of chronic disease; CCAI, Clinical Colitis Activity Index; CD, Crohn's disease; CDAI, Crohn's Disease Activity Index; CRP, C‐reactive protein; ESR, erythrocyte sedimentation rate; IDA, iron deficiency anemia; MTWAI, Modified Truelove and Witts activity index; NR, not reported; PCDAI, Pediatric Crohn's Disease Activity Index; PUCAI, Pediatric Ulcerative Colitis Activity Index; SCCAI, Simple Clinical Colitis Activity Index; UC, ulcerative colitis; UCAI, Ulcerative Colitis Activity Index.

### 3.2 Meta‐analysis of mean serum hepcidin levels

**FIGURE 2:**
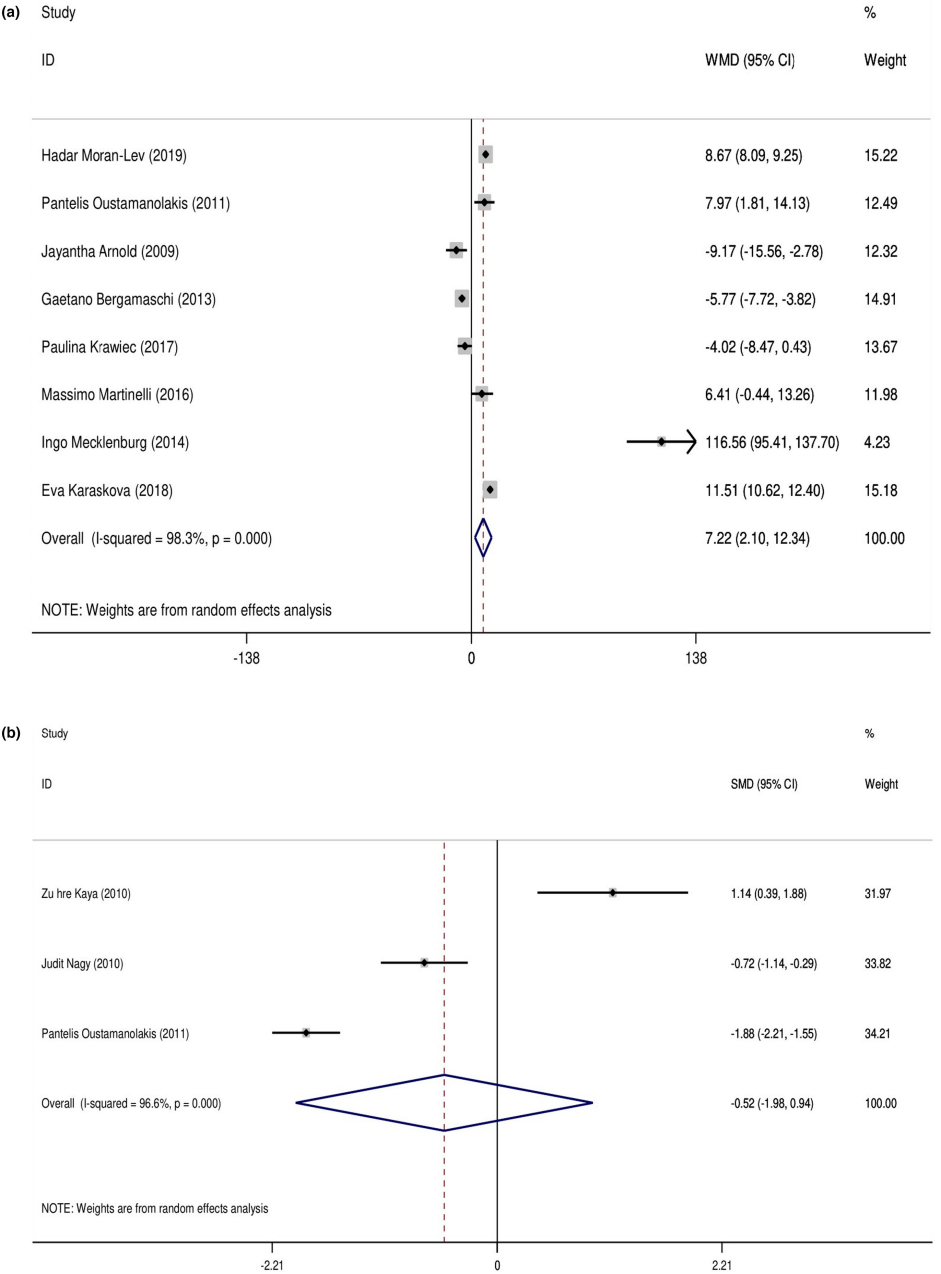
(a) Forest plot shows the weighted mean difference (WMD) in serum hepcidin concentrations between participants with IBD and healthy control participants. (b) Forest plot show the standardized mean difference (SMD) in serum prohepcidin concentrations between participants with IBD and healthy control participants.

Eight studies with 1021 participants (case = 701 and control = 320) reported the mean and SD of serum hepcidin levels in both IBD and control subjects. The random‐effects meta‐analysis revealed that subjects with IBD had 7.22 ng/mL (95% CI: 2.10, 12.34; *p =* .006) higher serum hepcidin concentrations compared with their healthy counterparts (Figure 2). However, the tests also showed that there was significant heterogeneity among the studies (*I* ^2^ = 98%, *p* < .001). We conducted subgroup analysis according to age, and the quality grade, in order to find possible sources of heterogeneity. These subgroup analyses did confirm that the quality scale represented a source of heterogeneity. Based on the findings of the age‐based subgroup analysis, IBD patients aged ≥18 years displayed increased levels of serum hepcidin versus healthy individuals (22.36 ng/mL, 95% CI, 2.12–42.61; *p =* .030; Table 2).

**TABLE 2:** Subgroup analysis based on the mean age of the participants and the quality of scale used.

	*N*	ES (95% CI)	*p*‐value, Significance test(s) for ES	*p*‐value, Intergroup Heterogeneity	*I* ^2^ (%)	*p*‐value, Heterogeneity between groups
Mean age						
<18	4	2.866 (0.490, 5.242)	.018	<.001	98	<.001
≥18	4	−0.073 (−1.093, 0.947)	.889	<.001	96	
Quality scale						
≥7	2	0.364 (0.136, 0.592)	.002	.937	0.0	<.001
≤6	6	1.721 (−0.099, 3.541)	.064	<.001	98	

Abbreviations: CI, confidence interval; ES, effect size; *I* ^2^, *I* ^2^ statistics; *p*‐value, level of significance.

### 3.3 Meta‐analysis of mean serum prohepcidin levels

Three studies with 365 participants (case = 217 and control = 148) reported the mean and SD of serum prohepcidin levels in both IBD and control subjects. Random‐effects meta‐analysis revealed that subjects with IBD had −0.522 ng/mL (95% CI: −1.983, 0.939; *p* = .484) nonsignificantly lower serum prohepcidin concentrations compared with their healthy counterparts (Figure 2). However, the tests also showed that there was significant heterogeneity among the studies (*I* ^2^ = 96%, *p* < .001).

### 3.4 Sensitivity analysis and publication bias

**FIGURE 3:**
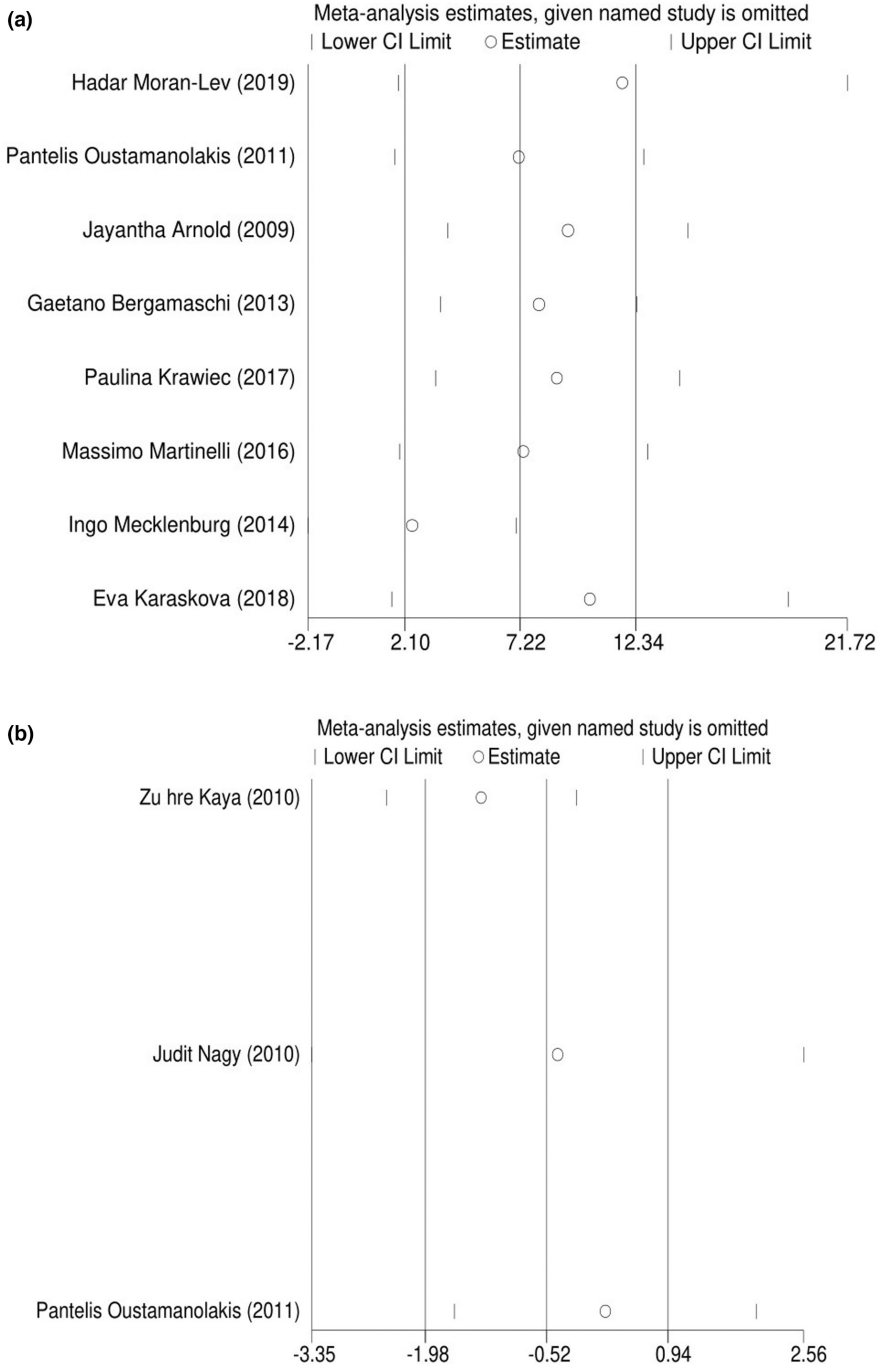
(a) Hepcidin leave‐one‐out sensitivity analysis. (b) Prohepcidin leave‐one‐out sensitivity analysis.

**FIGURE 4:**
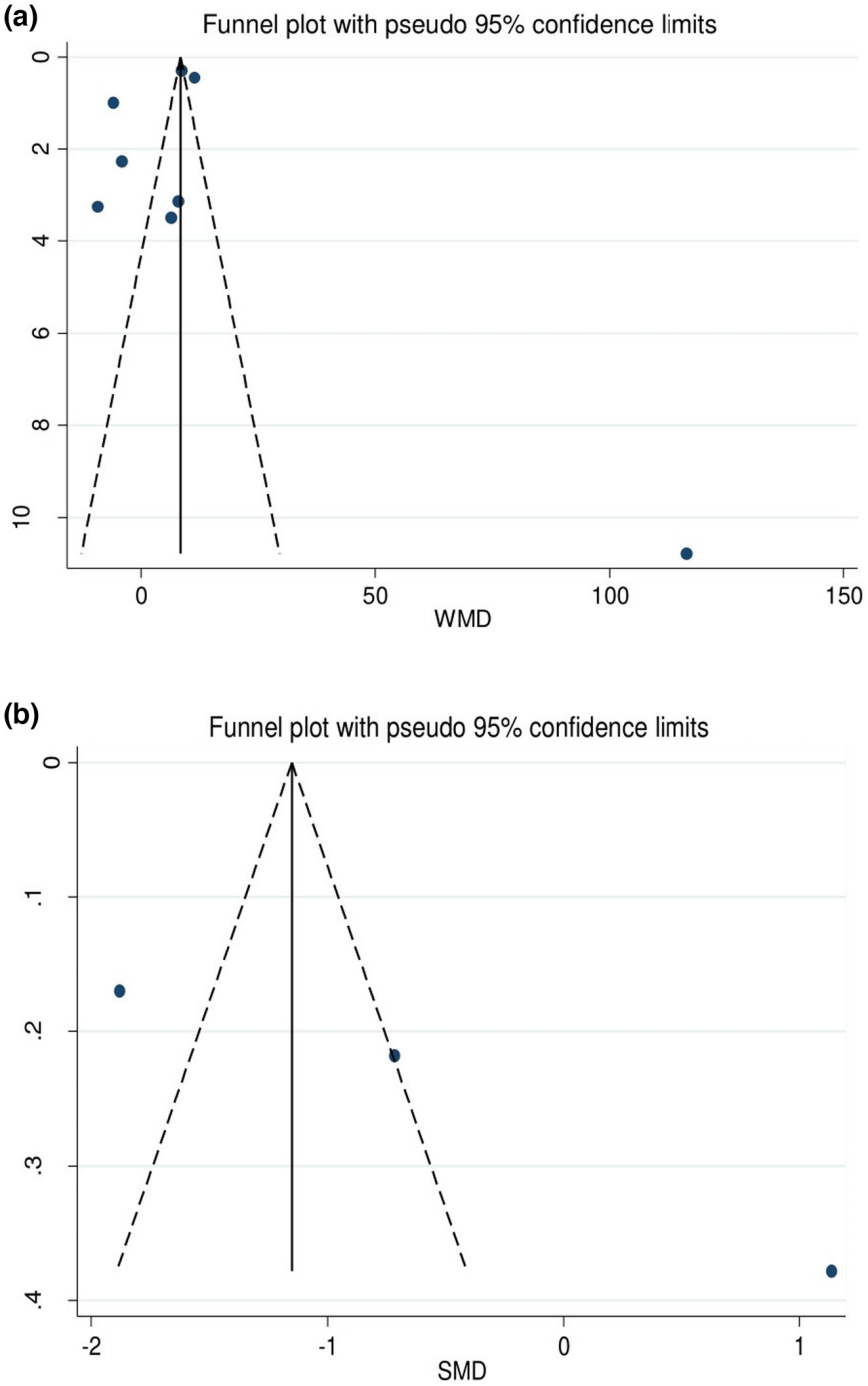
(a) Funnel plot of the weighted mean difference (WMD) versus the s.e. of the weighted mean difference (WMD) for hepcidin. (b) Funnel plot of the standardized mean difference (SMD) versus the s.e. of the standardized mean difference (SMD) for prohepcidin.

Sensitivity analysis revealed that removing some of the studies would not exert a significant impact on the overall results (Figure 3). Visual inspection of the funnel plot and Egger's test (hepcidin: *p* = .644, prohepcidin: *p* = .146) did not demonstrate evidence of publication bias in the meta‐analysis (Figure 4). The ‘trim‐and‐fill’ test estimated the effect of unpublished studies for prohepcidin levels (*n* = 5, WMD: −1.88 ng/mL, 95% CI: −3.34 to −0.41, *p* = .012).

## 4 DISCUSSION

In this systematic review and meta‐analysis, we observed that IBD patients had higher serum levels of hepcidin compared to healthy controls. Using data derived from eight studies with a total of 1021 participants (case = 701 and control = 320), the random‐effects meta‐analysis revealed that individuals diagnosed with IBD had 7.22 ng/mL (95% CI: 2.10, 12.34; *p* = .006) higher serum hepcidin concentrations. The reported reference range for serum hepcidin levels was 29–254 ng/mL in men (*n* = 65) and 17–286 ng/mL in women (*n* = 49), with median levels of 112 and 65 ng/mL, respectively (Ganz et al., 2008).

In addition to IBD, the studies mixed subjects with or without anemia. IBD encompassed both CD and UC in all studies except for one that did not report the type of IBD. We did not carry out specific analyses according to the type of IBD and presence/type of anemia due to limited sample size. At least when reported, we described the type of IBD and anemia in Table 1 as a means of showing further individual details. Taken together, hepcidin levels vary clinically across IBD. Mecklenburg et al. reported that hepcidin concentrations varied between IBD patients with active and/or inactive disease based on the presence of anemia and iron deficiency and that hepcidin values correlated only with ferritin levels and not with other inflammation markers' concentrations. For example, hepcidin levels were highest among subjects with active CD, anemia, and no iron deficiency (557 ng/mL), followed by patients with inactive CD, no anemia and no iron deficiency (457 ng/mL), and patients with active CD, no anemia, and no iron deficiency (388 ng/mL). Hepcidin levels were low in individuals with CD and anemia with subsequent iron deficiency (93–97 ng/mL). Similarly, UC patients with active disease, no iron deficiency with (698 ng/mL) or without anemia (577 ng/mL), and UC patients with inactive disease and without iron deficiency or anemia (464 ng/mL) had the highest hepcidin levels, whereas UC subjects with anemia, iron deficiency, and with/without active disease had lower hepcidin concentrations (16–54 ng/mL) (Mecklenburg et al., 2014). It is important to note that mean hepcidin levels remained above the proposed ranges (Ganz et al., 2008) even in the absence of anemia, shedding light on the relevance of screening hepcidin and ferritin levels in patients with IBD.

Some seminal data report an anemia prevalence of 6%–74% in IBD (Kulnigg & Gasche, 2006). Moreover, a meta‐analysis depicts a prevalence of anemia of 27% in CD and 21% in UC, with 57% of anemia cases being iron deficiency anemia (IDA; Filmann et al., 2014). IBD can be associated with both IDA and anemia of chronic disease (ACD), also known as anemia of inflammation (Karaskova et al., 2021). More prevalent in IBD, IDA is caused by chronic blood loss in the gastrointestinal tract from ulcerations, decreased iron intake, as well as defective iron absorption resulting from inflammation of the duodenum and upper jejunum mucosa (Murawska et al., 2016). Regarding ACD, it is a type of normocytic normochromic anemia that arises from inflammatory conditions whereby the IL‐6–hepcidin axis is triggered, leading to low serum iron levels but maintaining sufficient iron stores (Camaschella et al., 2019; Karaskova et al., 2021; Nemeth & Ganz, 2014).

There is a tight relationship between inflammation and the decrease in serum iron levels due to reduced intestinal iron absorption and increased iron retention in macrophages (Ganz, 2011). Synthesized in the liver under inflammatory stimuli, particularly induced by the IL‐6, hepcidin is a key mediator of this pathway and inhibits the iron flow from macrophages into circulation (Ganz, 2006). IL‐6 displays a pivotal role in human hepcidin synthesis, as shown by Nemeth et al. who detected 7.5‐fold higher urinary hepcidin concentrations 2 h after hepcidin infusion, alongside decreases of 34% and 33% in serum iron levels and transferrin saturation, respectively (Nemeth et al., 2004). In addition to IL‐6, hepcidin synthesis is mediated via its receptor and the signal transducer and activator of transcription‐3 (Rogler & Vavricka, 2014). Moreover, Basseri et al. found positive correlations between serum hepcidin and IL‐6 levels (*r* = .546, *p* = .023) and negative correlations between serum hepcidin and hemoglobin concentrations (*r* = .528, *p* = .029) in subjects with CD and ACD (Basseri et al., 2013).

Age‐based subgroup analyses pointed out that IBD patients aged ≥18 years displayed notable increases in serum hepcidin levels equivalent to 22.36 ng/mL (95% CI, 2.12, 42.61; *p =* .030) when compared to healthy individuals. In addition, patients <18 years with IBD had elevated (+6.66 ng/mL; 95% CI, 3.39–9.93; *p* < .001) serum hepcidin levels versus healthy individuals.

Among the studies that recruited patients aged <18 years, Martinelli et al (Martinelli et al., 2016) detected higher serum hepcidin levels in pediatric patients with active IBD when compared to healthy patients (9.4 ± 15.8 nM [0.55–49.2] vs. 2.1 ± 2.6 nM [0.55–11.3], *p* = .003). Such a result is of clinical relevance, since, from a general analysis of approximately 3000 patients, a mean hepcidin concentration of 7.8 nM was found in men and 4.1 nM versus 8.5 nM for premenopausal and postmenopausal women, respectively (Galesloot et al., 2011). Karaskova et al. highlighted that children with newly diagnosed with CD (*n* = 53) had significantly higher serum hepcidin levels compared to subjects with UC (*n* = 23), that is, 22.6 ng/mL (8.5–65.0) versus 6.5 ng/mL (2.4–25.8) (*p* < .05) (Karaskova et al., 2018). In children with IBD (*n* = 34), Wiskin et al. (2012) detected an anemia prevalence of 75% at diagnosis and 30% at follow‐up 2 years later.

Notwithstanding, attention to iron administration is fundamental in this scenario, as hepcidin expression is directly regulated by the iron intake and its downregulation increases the bioavailability of circulating iron levels (Daher et al., 2017). Nevertheless, given that IBD patients have lower intestinal iron absorption, intravenous iron administration has greater efficacy in increasing hemoglobin and ferritin levels compared to oral iron administration, as shown by several meta‐analyses (Bonovas et al., 2016; Lee et al., 2012). Interestingly, serum hepcidin levels are regarded as a good predictor of iron malabsorption in IBD patients, with sensitivity and specificity at levels >95% for the oral iron absorption test (Aksan et al., 2019). In addition, serum hepcidin levels tend to predict iron malabsorption slightly better than serum ferritin concentrations (Aksan et al., 2019).

Regarding disease activity, Oustamanolakis et al. observed a positive correlation between hepcidin (but not prohepcidin) levels and disease status in patients suffering from UC (11). Interestingly, Mecklenburg et al. observed decreased hepcidin serum levels in anemic IBD patients with depleted iron storage regardless of disease activity status (24). In children with IBD, while one study found a positive correlation between increased levels of serum hepcidin and disease activity state (22), another did not (13).

Apart from hepcidin, we found nonsignificant (−0.522 ng/mL; 95% CI: −1.983 to 0.939; *p* = .484) lower serum prohepcidin concentrations compared to healthy counterparts. It must be noted, however, that the analysis was based on only three studies. A better understanding of the prohepcidin role in IBD may allow more clues for managing this disease. Inflammation is a key player in IBD and several assessments have highlighted that anti‐TNF monoclonal antibodies, namely, infliximab and adalimumab, are effective therapeutic options in inducing remission in moderate to severe IBD by downregulating several pro‐inflammatory mediators (Cavallaro et al., 2017). More specifically, after six weeks of anti‐TNF administration in IBD patients, serum prohepcidin, as well as IL‐6, C‐reactive protein, and ferritin concentrations decreased significantly, while serum iron, hemoglobin, and total transferrin levels increased (Cavallaro et al., 2017). In addition, Shu et al have highlighted that hepcidin concentrations are elevated in individuals with active disease versus IBD in remission and that hepcidin values correlate with IBD activity and severity of anemia, as well as with erythrocyte sedimentation rate (ESR), CRP, TNF‐alpha, IL‐6, and IL‐17 values (Shu et al., 2019). Interestingly, their assessment also demonstrated that the administration of infliximab, an anti‐TNF‐alpha monoclonal antibody, was linked with reduced hepcidin levels due to its inhibitory impact on the upregulation of hepcidin mRNA which seems to be dependent on the NF‐κB and caspase‐3/8 pathways. Consequently, prescription of infliximab is believed to improve anemia in IBD individuals and we may, thus, hypothesize that patients with elevated hepcidin values and anemia might benefit more from the use of these drugs and be selected as appropriate candidates for the prescription of this monoclonal antibody (Shu et al., 2019). Moreover, investigations using murine models of IBD have pointed out that gut inflammation and bone morphogenetic proteins enhance expression of hepcidin and that the use of pharmacological agents that target bone morphogenetic proteins, for example, agents that prevent the binding of these molecules to their receptors, suppress signal transduction, or act as antibodies against these molecules, results in improved iron availability and decreased hepcidin levels (Wang et al., 2012). Similarly, Loveikyte et al have revealed that evaluation of hepcidin concentrations is of paramount importance in IBD since its levels can aid in the differentiation of iron deficiency and functional iron restriction even when IBD is associated with elevated inflammation levels (Loveikyte et al., 2023). In their study, infliximab or vedolizumab induction‐based treatment decreased hepcidin, ferritin, and inflammation markers' values; however, this effect was only noted in treatment responders. Therefore, measurement of hepcidin concentrations in IBD might be of relevance to distinguish individuals who are likely to benefit from monoclonal antibodies versus those who will not and would require other therapeutic regimens (Loveikyte et al., 2023). Furthermore, as IDA is a notable complication in IBD, patients with CD and/or UC often require iron replacement therapies. Hepcidin evaluation is useful in such instances, as Lalosevic et al have discovered that assessment of hepcidin concentrations can help identify IBD subjects with IDA (Stojkovic Lalosevic et al., 2020). Moreover, Aksan et al. have demonstrated that there is a negative association of hepcidin levels and serum iron concentrations in individuals with IBD who have been prescribed oral iron supplements (Aksan et al., 2020). Thus, the findings of our meta‐analysis can be used to predict which IBD patients might be poor responders to oral iron supplementation, as an elevation in hepcidin concentrations seems to be linked with a decrease in intestinal absorption of iron. In addition, it has been shown that hepcidin is involved in the pathophysiological link that leads to the decrease in the synthesis erythropoietin and leads to its impaired biological activity which can further be counteracted by the anti‐TNF‐alpha activity of infliximab. Nevertheless, hepcidin are not only elevated in ACD but also in mixed types of anemia, that is, a combination of IDA and ACD that can be detected in IBD (Guagnozzi & Lucendo, 2014). Thus, even a slight elevation in hepcidin could impact the formation of red blood cells in IBD.

Since transferrin is a blood plasma glycoprotein with a high affinity for ferric iron and hence transports iron through the circulating to many tissues (e.g., liver, spleen, and bone marrow) (Ogun & Adeyinka, 2018), a lower transferrin saturation can be observed in IBD, as supported by some studies (Bergamaschi et al., 2013; Krawiec et al., 2017; Mecklenburg et al., 2014). In contrast, no correlation between transferrin saturation and IBD was observed in other studies (Martinelli et al., 2016; Oustamanolakis et al., 2011).

Indeed, measurement of serum hepcidin is a useful complementary therapeutic tool for screening IBD‐related anemia, as there is a strong relationship between hepcidin and iron absorption (Ganz & Nemeth, 2012), such that anemia is a poor prognostic factor and diminishes patients' long‐term quality of life (Bailey et al., 2012). Thus, our results are of great importance in terms of providing a skeptical view regarding changes in hepcidin levels in IBD patients. However, the clinical situation differs depending on the types of anemia and IBD and thus requires a personalized decision‐making practice.

Regarding the limitations of our research, we highlight the highly significant interstudy heterogeneity in terms of hepcidin and prohepcidin levels (*I* ^2^ = 97%, *p* < .001; *I* ^2^ = 96%, *p* < .001, respectively). Although we performed funnel plots and Egger's linear regression tests in an attempt to investigate basic publication biases, particular clinical flaws inherent to the studies can persist. Besides, given that we did not perform analyses based on the types of IBD and presence or absence of anemia due to the limited sample size and underreporting, further research is warranted in this regard. Another limitation of our research is that hepcidin and prohepcidin concentrations might vary based on different laboratories, techniques used, and time from sample collection to final analysis. Most of the analyzed investigations collected serum samples and measured hepcidin and/or prohepcidin using ELISA, although some employed radioimmunoassays, mass spectrometry, or liquid chromatography. For example, in Mecklenburg et al.'s study, the measurement range for hepcidin was relatively wide, with some patients with active disease displaying hepcidin concentrations of 2125 ng/mL. Some factors which could have contributed to these discrepancies are that the study included a higher number of patients with IBD and anemia and that due to small sample sizes, during the statistical analysis of the data, the researchers combined CD and UC patients together in order to report hepcidin levels (Mecklenburg et al., 2014).

As serum hepcidin levels may better predict iron malabsorption than serum ferritin (Aksan et al., 2019), ordering hepcidin levels can be feasible to improve decision‐making regarding oral versus intravenous iron administration. In addition to common IBD‐related anemia, perhaps screening for hepcidin levels could be useful in more severe IBD associated with blood transfusion and cancer. Ultimately, further epidemiological and clinical studies are needed to clarify the extent to which changes in hepcidin levels affect IBD patients.

## 5 CONCLUSION

IBD patients had higher serum levels of hepcidin when compared to healthy control groups. However, caution should be exercised when translating our findings toward real‐world intervention, insofar as clinical variation in hepcidin status remains to be fully elucidated in CD and UC.

## AUTHOR CONTRIBUTIONS

**Samira Soltanieh:** Conceptualization (equal); data curation (equal); investigation (equal); methodology (equal); project administration (equal); validation (equal). **Marieh Salavatizadeh:** Methodology (equal); software (equal); writing – original draft (equal). **Mihnea‐Alexandru Gaman:** Writing – original draft (equal); writing – review and editing (equal). **Hamed Kord Varkaneh:** Formal analysis (equal); software (equal). **Shing Cheng Tan:** Conceptualization (equal); methodology (equal); writing – review and editing (equal). **Kousalya Prabahar:** Resources (equal); writing – original draft (equal). **Oana Deliu Lozovanu:** Data curation (equal); formal analysis (equal); investigation (equal); visualization (equal). **Heitor O. Santos:** Writing – original draft (equal). **Azita Hekmatdoost:** Conceptualization (equal); software (equal); supervision (equal); validation (equal); writing – original draft (equal).

## FUNDING INFORMATION

Shahid Beheshti University of Medical Sciences.

## CONFLICT OF INTEREST STATEMENT

The authors declare that they have no conflict of interest.

## ETHICS APPROVAL AND CONSENT TO PARTICIPATE

The study protocol was approved by the Ethics Committee of the National Nutrition and Food Technology Research Institute at Shahid Beheshti University of Medical Sciences.

## Data Availability

The datasets generated during the current study are available from the corresponding author upon reasonable request.
